# Upregulation of Enzymes involved in ISGylation and Ubiquitination in patients with hepatocellular carcinoma

**DOI:** 10.7150/ijms.39823

**Published:** 2020-01-20

**Authors:** Hoang Van Tong, Nghiem Xuan Hoan, Mai Thanh Binh, Dao Thanh Quyen, Christian G. Meyer, Dinh Thi Thu Hang, Dinh Thi Dieu Hang, Ho Anh Son, Hoang Van Luong, Nghiem Duc Thuan, Nguyen Truong Giang, Do Quyet, Mai Hong Bang, Le Huu Song, Thirumalaisamy P. Velavan, Nguyen Linh Toan

**Affiliations:** 1 Institute of Biomedicine and Pharmacy, Vietnam Military Medical University, Hanoi, Vietnam; 2 Department of Pathophysiology, Vietnam Military Medical University, Hanoi, Vietnam; 3 108 Military Central Hospital, Hanoi, Vietnam; 4 Vietnamese-German Center of Excellence in Medical Research, Hanoi, Vietnam; 5 Institute of Tropical Medicine, University of Tübingen, Tübingen, Germany; 6 Duy Tan University, Da Nang, Vietnam; 7 Hai Duong Medical Technical University, Hai Duong, Vietnam

**Keywords:** hepatocellular carcinoma, Interferon-stimulated gene 15 (ISG15), ISGylation, E3 ligase, ubiquitin-specific protease 18 (USP18)

## Abstract

**Background**: ISGylation is the conjugation of ISG15 with target proteins. ISGylation occurs through an enzymatic cascade, which is similar to that of ubiquitination. Through ISGylation, ISG15 can bind to proteins involved in cell proliferation and differentiation, thus promoting genesis and progression of malignancies. The present study aims to investigate expression of genes involved in ISGylation and ubiquitination in patients with hepatocellular carcinoma and to correlate gene expression with clinical laboratory parameters of these patients.

**Methods**: mRNA expression of genes encoding enzymes involved in the ISGylation process (*EFP*, *HERC5, UBA1, UBC* and *USP18*) was evaluated by quantitative real-time PCR in 38 pairs of tumour and adjacent non-tumour tissues from patients with hepatocellular carcinoma and correlated with distinct clinical laboratory parameters.

**Results:** Relative mRNA expression of *EFP*, *HERC5, UBA1* and *USP18* was significantly higher in tumour tissues compared to adjacent non-tumour tissues (*P*=0.006; 0.012; 0.02 and 0.039, respectively). The correlation pattern of mRNA expression between genes in the tumours differed from the pattern in adjacent non-tumour tissues. Relative expression of *EFP*, *HERC5* and *UBA1* in adjacent non-tumour tissues was positively associated with direct bilirubin levels (Spearman's rho=0.31, 0.33 and 0.45; *P*=0.06, 0.05 and 0.01, respectively) and relative expression of *USP18* in adjacent non-tumour tissues correlated negatively with ALT levels (Spearman's rho= -0.33, *P*=0.03).

**Conclusions**: *EFP*, *HERC5, UBA1,* and *USP18* genes are upregulated in tumour tissues of patients with HCC and, thus, may be associated with the pathogenesis of hepatocellular carcinoma.

## Introduction

Hepatitis B virus (HBV) infection is responsible for up to 90% of hepatocellular carcinoma (HCC) cases, especially in regions with a high prevalence of HBV infection including Vietnam 1, 2. HCC accounts for 75-85% of primary liver cancer and is the fourth leading cause of cancer-related deaths. Annually, 841,000 new cases and 782,000 deaths are reported worldwide 2. The number of HCC-related deaths is increasing and estimated to reach a prevalence of one million by 2030 3. HBV infection can cause HCC through direct interaction of viral factors (e.g. HBV-DNA, HBx protein) with host cells, causing instability of human chromosomes and induction of oncogenes. Indirect mechanisms result from inflammation of hepatocytes, fibrosis and modification of cellular signalling involving the JAK/STAT and Wnt/β-catenin pathways 4.

In response to HBV infection, immune cells secrete interferon (IFN) and other inflammatory cytokines. Subsequently, cellular signalling pathways are activated and IFN-stimulated genes (ISGs) are induced 5. Among the ISGs, ISG15 is a key effector molecule of the innate immune system. ISG15 is induced by type I interferon and lipopolysaccharides and has antiviral activity against many viruses 6. It plays also an important role in several immunomodulatory activities, including induction of NK cell proliferation, triggering T cell responses, augmentation of lymphokine-activated killer (LAK) activity, stimulation of IFN-γ production, inducing dendritic cell (DC) maturation and acting as a chemotactic factor for neutrophils 7, 8. ISG15 exists both as a free form and/or in conjugation with other proteins. The conjugation of ISG15, ISGylation, occurs through an enzymatic cascade through which ISG15 can modulate the functions of pathogenic and host proteins and affect downstream signalling pathways 9. Initially, the activation of the E1 enzyme (ubiquitin-like modifier-activating enzyme 7; UBA1 or UbE1L) forms a thioester bond with free ISG15 in an ATP-dependent manner 7. ISG15 is then transferred to the ubiquitin/ISG15-conjugating enzyme E2 L6 (UBE2L6 or UbcH8). Finally, E3 ligases catalyze the conjugation of ISG15 with lysine residues of target substrate proteins, a process which may be reversible (deISGylation) by the activity of the ubiquitin-specific protease 18 (USP18) 6.

ISGylation has multiple functions, including inhibition of release of exosomes, downregulation of protein translation and regulation of distinct protein functions 10. In cancer, ISGylation has been shown to target p53, a tumour protein, and control its stability and functionality 11. Through ISGylation, ISG15 is associated with the formation, development and progression of several malignancies such as breast, lung and ovarian cancers 12-14. Several enzymes involved in the ISGylation process are associated with cancers in both an ISGylation-dependent and ISGylation-independent manner. Particularly, the HECT domain and the RCC1-like domain-containing protein 5 (HERC5) and estrogen-responsive finger protein (EFP or TRIM25), known as E3 ligase of ISGylation have been shown to be associated with liver, breast prostate, endometrial and ovarian cancers 15-18. USP18 is also involved in the development of breast, lung and liver cancers 19-21. A study has indicated that USP18 contributes to controlling carcinogenesis, as loss of USP18 function increases apoptosis and decreases cell proliferation by destabilization of the cyclin D1 protein 22.

It remains, however, unclear so far whether the *EFP*, *HERC5* and *USP18* genes contribute to tumourigenesis through ISG15/ISGylation or other mechanisms. In addition, the exact role of ISG15 and other key enzymes involved in ISGylation in the formation and development of HBV-related HCC is still unclear. In the present study, we aim to investigate the expression profiles of genes encoding enzymes involved in the ISGylation process (*EFP*, *HERC5, UBA1, UBC USP18*) in patients with HCC and their association with clinical outcomes.

## Methods

### Study subjects

The study included 38 patients with HCC who underwent surgery at the 108 Military Central Hospital in Hanoi, Vietnam. The patients were diagnosed according to the guidelines of the American Association for the Study of Liver Diseases (AASLD) for management of HCC 21. HCC was confirmed by computed tomography (CT) imaging and/or by histology and classified based on the Barcelona Clinic Liver Cancer (BCLC) classification system 26. HBV and HCV infections were assessed in order to determine the etiology of the disease. Liver tissue specimens collected from the HCC patients included tumour and adjacent non-tumour specimens; the samples were and frozen at -80^0^C until further use. Patients were further grouped as Child-A, Child-B or Child-C according to Child-Pugh scores 22. Laboratory parameters involving blood counts, total and direct bilirubin, prothrombin, albumin, alanine transaminase (ALT), aspartate transaminase (AST), and the tumour marker alpha-fetoprotein (AFP) were assessed by routine laboratory tests. The baseline characteristics of the 38 HCC patients including age, gender and the laboratory results are shown in Table 1.

All details of the study were explained to the participants and informed written consent was obtained before taking samples. All clinical procedures and experiments were performed in accordance with applying guidelines and regulations. The study was approved by the Institutional Review Board of the 108 Central Hospital, Hanoi, Vietnam.

### Quantification of relative gene expression by quantitative real-time PCR

Total RNA was extracted from the 38 tumour and adjacent non-tumour tissue pairs by Trizol reagent (Life Technologies, Carlsbad, CA, USA) according to the protocol recommended by the manufacturer. Extracted total RNA samples were reverse transcribed into cDNA by using the QuantiTect Reverse Transcription Kit (Qiagen, Hilden, Germany). cDNA was quantified by real-time PCR with *GAPDH* (glyceraldehyde-3-phosphate dehydrogenase) used as reference gene. Sequences of primers used in this study are presented in Table 2. Quantitative realtime PCR reactions were carried out in a volume of 25 μl containing 12.5 μl 2x SYBR Green PCR master mix (Bioline, Luckenwalde, Germany), 0.5 µM primer pairs specific for the target genes and the reference gene, 5 ng cDNA samples and RNase-free water up to the 25 μl reaction volume. Thermal cycling conditions were 95 °C for 2 minutes for initial activation, followed by 45 cycles of denaturation at 95 °C for 5 sec and annealing and extension at 58 °C for 20 sec. Melting curve analyses starting from 58 °C to 85 °C were performed after each run to confirm specificity of the PCR products. All reactions were performed in duplicates and repeated twice using the LightCycler^®^ 480 real-time PCR system (Roche, Basel, Switzerland). Relative expression of target genes was normalized to the expression of *GAPDH* based on the ΔCt method.

### Statistical analysis

Quantitative parameters including laboratory tests and relative gene expression values are given as means with standard deviation or medians with ranges where appropriate. The relative gene expression between tumour and adjacent non-tumour tissues was compared using the Wilcoxon signed ranks test. Spearman's rank correlation coefficient was used to analyze the correlation of relative gene expression between two genes or between expression of the genes with clinical parameters. The SPSS software version 22.0 (SPSS Statistics, IBM, Armonk, NY, the USA) was used for all statistical analyses; the significance level was set at *P<*0.05.

## Results

### Gene expression in HCC patients

**Figure 1:**
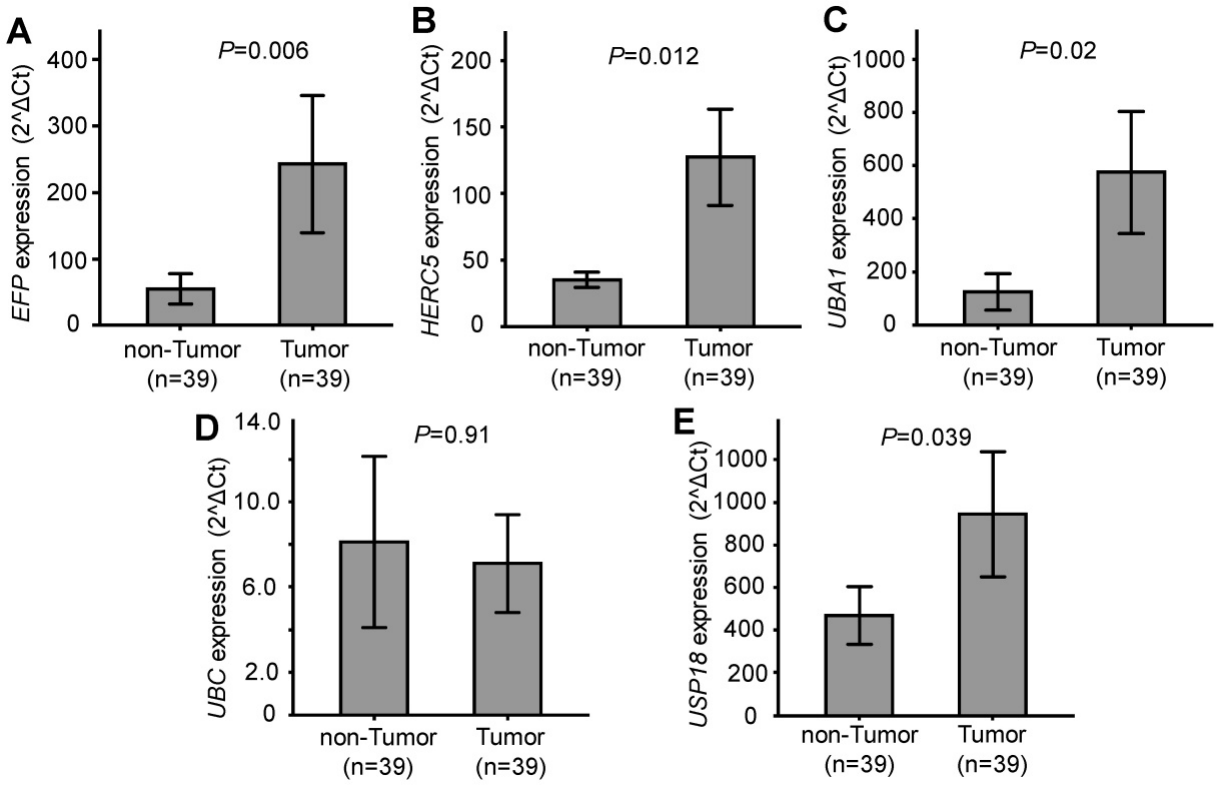
** Gene expression in tumour and adjacent non-tumour tissues.** A, B, C, D and E: Relative expression of *EFP*, *HERC5, UBA1, UBC* and *USP18,* respectively.* P* values were calculated by Wilcoxon signed ranks test.

Relative expression of the five genes *EFP*, *HERC5, UBA1, UBC* and *USP18,* which are related to ISGylation, was quantified and compared between HCC tumour and adjacent non-tumour tissues. Expression of *EFP*, *HERC5, UBA1,* and *USP18* was significantly higher in HCC tumour tissues compared to the adjacent non-tumour tissues (*P*=0.006, 0.012, 0.02 and 0.039, respectively). No significant difference of *UBC* expression was observed between HCC tumour tissues and non-tumour tissues (*P*=0.91) (Figure 1). These results indicate that *EFP*, *HERC5, UBA1,* and *USP18* are upregulated in HCC tumours and might be associated with the development of HCC.

We then examined whether gene expression was associated with progression of HCC by comparing their expression between different BCLC stages. However, expression of the genes did not differ between stage A and B HCC tissues as well as between the corresponding non-tumour tissues (*P*>0.05). BCLC stages C and D did not occur among the study participants.

### Correlation of mRNA expression between genes

We analysed the correlations of mRNA expression in both HCC tumour and non-tumour tissues. In HCC tissues, gene expression was strongly correlated with each other (Spearman's rho >0.85; *P*<0.0001). The strongest correlation was observed between expression of *EFP* and *HERC5* (Spearman's rho =0.98; *P*<0.0001), followed by expression of *HERC5* and *USP18* (Spearman's rho=0.95; *P*<0.0001) and of *EFP* and *UBA1* (Spearman's rho =0.94; *P*<0.0001) (Table 3). In non-tumour tissues, *EFP* expression was strongly correlated with that of *HERC5*, *UBA1* and *USP18* (Spearman's rho =0.81, 0.89 and 0.75; *P*<0.0001, respectively). A similar result was observed when the correlation of *HERC5* expression with that of *UBA1* and *USP18* was analysed (Spearman's rho=0.76; *P<*0.0001) (Table 3). The correlation of *UBC* mRNA expression with *EFP*, *UBA1* and *USP18* was moderately positive (Spearman's rho =0.58, 49 and 54; *P<*0.0001, 0.003 and 0.0007, respectively). A similar correlation was seen between *UBA1* and *USP18* (Spearman's rho =0.56; *P=*0.0005). These results indicate that the expression pattern of the genes under study differed between HCC tumour and adjacent non-tumour tissues.

### Correlation of gene expression with laboratory parameters

**Figure 2:**
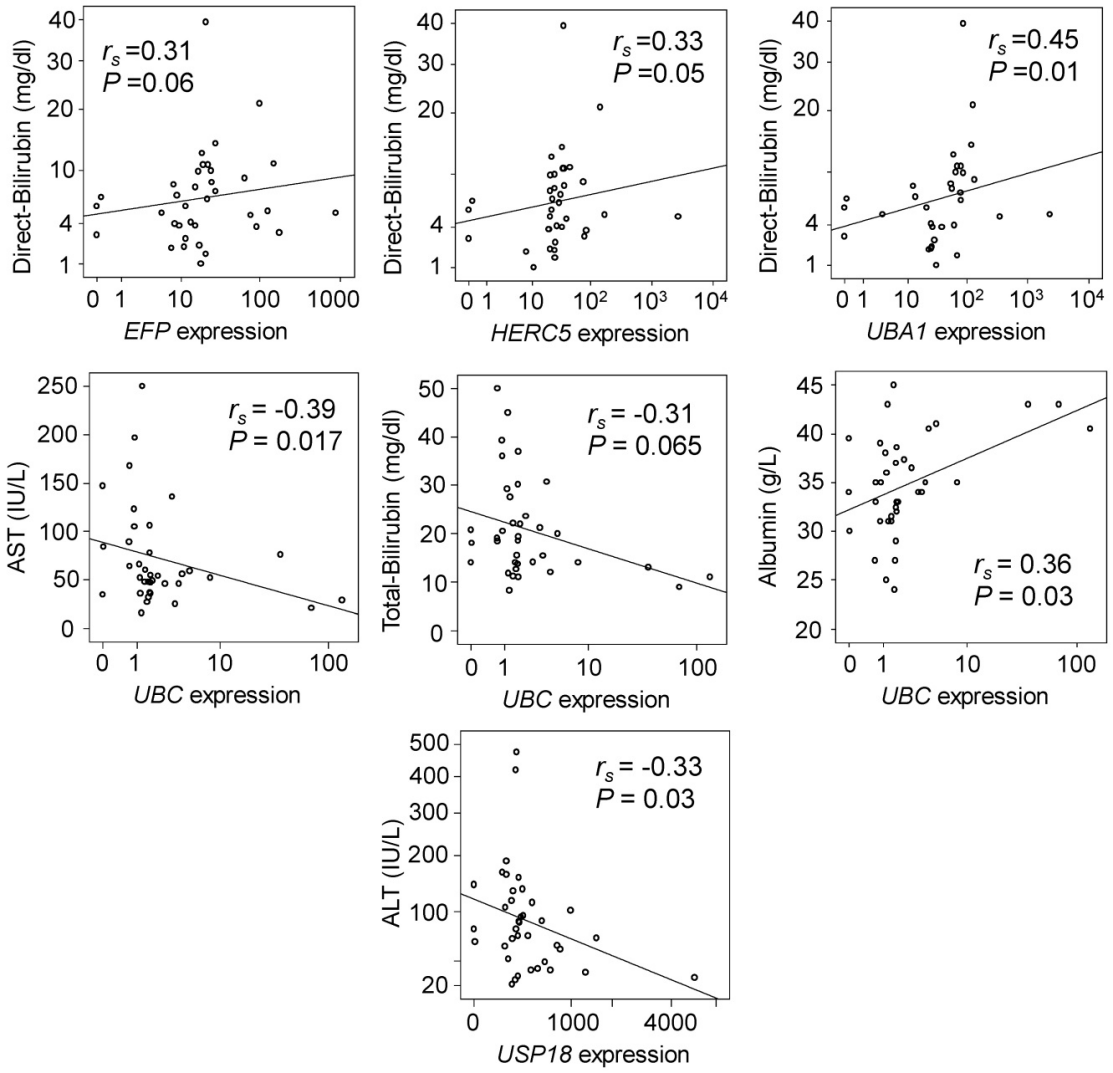
** Correlation of gene expression with clinical parameters.** Spearman's rho (r_s_) and *P* values presented in the figures were calculated by Spearman's rank correlation coefficient.

We analysed the correlations of relative gene expression with laboratory parameters of HCC patients. Expression of *EFP*, *HERC5* and *UBA1* in non-tumour tissues were positively correlated with direct bilirubin levels (Spearman's rho =0.31, 0.33 and 0.45; *P*=0.06, 0.05 and 0.01, respectively) (Figure 2, upper panel). Expression of *UBC* in non-tumour tissues was negatively correlated with AST and total bilirubin levels (Spearman's rho= -0.39 and -0.31; *P*=0.017 and 0.065, respectively), but positively correlated with albumin levels (Spearman's rho= 0.36; *P*=0.03) (Figure 2, middle panel). In addition, expression of USP18 in non-tumour tissues was negatively correlated with ALT levels (Spearman's rho= -0.33, *P*=0.03) (Figure 2, lower panel). However, in HCC tumour tissues no significant correlation was observed.

## Discussion

ISGylation plays a key role both in immunity against infections and in the formation and progression of tumours by affecting a wide range of target proteins 10. A number of enzymes involved in the ISGylation process, namely E3 ligases (HERC5 and EFP/TRIM25) and USP18 have been shown to be associated with cancers via ISGylation-dependent and -independent mechanisms 15-18. We have previously shown that *ISG15* expression is associated with HBV-related liver diseases, including HCC 23. Here we show that expression of E3 ligases (*HERC5* and *EFP/TRIM25*), *UBA1* and *USP18* is significantly upregulated in HCC tumours compared to adjacent non-tumour liver tissues. In addition, the expression levels of the genes in non-tumour tissues is associated with several clinical parameters. Our results indicate that E3 ligases, *UBA1* and *USP18* are associated with HCC development and may possibly be considered as targets in the treatment of HCC.

The upregulation of *EFP*, *HERC5*, *UBA1* and *USP18* in HCC tissues and the different patterns of correlation of the genes in HCC tumour and in adjacent non-tumour tissues suggest that these genes may play a role in the pathogenesis of HCC. Studies have shown that *EFP* is closely associated with breast cancer 6, 24. *EFP* regulates metastasis of breast cancer through its E3 ubiquitin ligase activity rather than through estrogen signalling 24. Expression of *EFP* is upregulated in breast and gastric cancers but downregulated in endometrial cancer 15, 25, 26. High *EFP* expression is associated with a poor prognosis of breast cancer 25, associated with advanced disease in human epithelial ovarian cancer 16, and high *EFP* expression is a prognostic factor in prostate cancer 26. It has also been shown that *EFP* increases proliferation and survival of prostate cancer cells by affecting p53 signals 26. *EFP* is also associated with HCC progression through degrading the metastasis-associated 1 protein (MTA-1) 27, 28. Here we show that *EFP* is significantly upregulated in HCC tissue compared to non-tumour liver tissues and *EFP* expression in non-tumour tissues is correlated with the levels of direct bilirubin.

HERC5 is the dominant E3 enzyme for ISGylation and has been shown to be highly expressed in HCC tissues and *in vitro* 18. It contributes to immune evasion in HCC and may be a prognostic biomarker for recurrent HCC after liver transplantation 17. In line with a previous study 18, we also observed upregulation of *HERC5* in HCC tumour tissues and a correlation of *HERC5* expression with direct bilirubin levels in non-tumour tissues. Although *UBA1* has been considered as a potential target gene for cancer therapy, especially for acute myeloid leukemia 29, the role of this gene in the pathogenesis of HCC had not been elucidated so far. To the best of our knowledge, this study for the first-time analyses *UBA1* expression in HCC tumour and adjacent non-tumour tissues and points towards its correlation with direct bilirubin levels.

In agreement with a previous report 21, our study also shows that *USP18* is significantly upregulated in HCC tissues compared to corresponding non-tumour tissues. USP18 is an important factor in the immune response directed against HBV replication and its overexpression promotes growth of HCC cells 21. Our results underline the crucial role of USP18 in HCC tumourigenesis; however, the exact mechanism by which USP18 is involved in HCC development remains unclear, as USP18 exhibits diverse functions. It influences cell cycle progression by interacting with S-phase kinase-associated protein 2 (SKP2) 30 and plays an important role in inhibition of HBV replication through activation of the JAK/STAT signalling pathway 31, independent of ISG15 and ISGylation 32. Recently, expression of *USP18* mRNA in peripheral blood mononuclear cells (PBMCs) has been proposed as a predictive factor for monitoring treatment of HBV patients with interferon-alpha 33. Another mechanism by which USP18 might contribute to tumour formation and progression in lung cancer is that USP18 can influence functionality of the cyclin D1 and KRAS proteins 20, 22. Although USP18 has been shown to inhibit tumourigenesis 34, further studies are required to describe in more detail the precise role of USP18 in HBV-related HCC.

*UBC* expression is upregulated in lung cancer 35. Although the relative expression of *UBC* does not differ significantly between HCC tumour and non-tumour tissues, *UBC* expression in non-tumour tissues is correlated with AST levels, total bilirubin and albumin, indicating that UBC may not significantly contribute to the pathogenesis of HCC, but rather plays a role in liver functions. Limitations of this study apply to the rather small number of HCC patients, particularly the small number of HCC patients in BCLC stages C and D, implying the impossibility to perform association analyses of gene expression with HCC progression.

In conclusion, our study shows that expression of *EFP*, *HERC5, UBA1* and *USP18* genes, upregulated in tumour tissues of HCC patients, is correlated with liver function parameters and, thus, may be associated with the pathogenesis of liver cancer.

## Figures and Tables

**Table 1:** Characteristics of 38 HCC patients

Characteristics	n (%)
**Age (years)**	
< 40	4/38 (10.5)
40 - 60	25/38 (65.8)
> 60	9/38 (23.7)
**Gender**	
Male	34/38 (89.5)
Female	4/38 (10.5)
**Etiology**	
HBV	29/38 (76.3)
HCV	1/38 (2.6)
Non-HBV/HCV	8/38 (21.1)
**BCLC staging Classification**	
Stage A	31/38 (81.6)
Stage B	7/38 (18.4)
Stage C and D	0/ 38 (0)
**Child Pugh Classification**	
Child A	18/38 (47.4)
Child B	6/38 (15.8)
Unclassified	14/38 (36.8)
**Clinical parameters**	**Median [Range]**
WBC (x10^6^/ml)	7.3 [3.88-15.4]
RBC (x10^3^/ml)	4.46 [2.7-5.8]
HCT (%)	131 [109-151]
PLT (10^3^/ml)	170.5 [97-497]
HBV-DNA	NA
AST (IU/ml)	54.5 [16 - 250]
ALT (IU/ml)	76 [21 - 476]
Total Bilirubin (µmol/l)	18.8 [8.2 - 50]
Direct Bilirubin (µmol/l)	5.7 [1 - 39.4]
Prothrombin (% of standard)	86 [50 - 125]
Protein (g/l)	65 [52 - 74]
Allbumin (g/l)	35 [24 - 45]
AFP (IU/ml)	45 [1 - 2479]

Abbreviation: BCLC, Barcelona Clinic Liver Cancer; HBV, HCV, hepatitis B and hepatitis C virus; HCC, hepatocellular carcinoma; AFP, alpha fetoprotein; WBC, white blood cells; RBC, red blood cells; HCT, hematocrit; PLT, platelets; AST and ALT, aspartate and alanine amino transferase; IU, international unit; NA, not applicable.

**Table 2:** Primers used for this study

Primer	Sequence	Target gene	Reference
*USP18_F*	5'- TGT CAG TCC ATC CTG GCT GAG TC -3'	*USP18*	This study
*USP18_R*	5'- CAC CTG AAT CAA GGA GTT AAG GCA GC -3'		
*UBA1_F*	5'- GCT CGC CGC TGT CCA AGA AAC -3'	*UBA1*	This study
*UBA1_R*	5'- GGG AGT AAA GGC CCT CGT CTA TGT C -3'		
*EFP_F*	5'- CGT GGA GTG GTT CAA CAC -3'	*EFP* (*TRIM25*)	25
*EFP_R*	5'- GAG CAG ATG GAG AGT GTG G -3'		
*HERC5_F*	5'- AAC CTG CAT GGG CAG CTT GG -3'	*HERC5*	36
*HERC5_R*	5'- TGT GGG CTT CTC CGG CAG AA -3'		
*UBC_F*	5'-GGG TCG CAG TTC TTG TTT GT-3'	*UBC*	35
*UBC_R*	5'-TCC AGC AAA GAT CAG CCT CT-3'		
*GAPDH_F*	5'- TGA ACG GGA AGC TCA CTG G -3'	*GADPH*	25
*GAPDH_R*	5'- TCC ACC ACC CTG TTG CTG TA-3'		

**Table 3:** Correlation of relative expression between genes in tumour and adjacent non-tumour tissues

Gene	*EFP*		*HERC5*		*UBA1*		*UBC*		*USP18*	
	*r_s_*	*P*	*r_s_*	*P*	*r_s_*	*P*	*r_s_*	*P*	*r_s_*	*P*
***EFP***			0.98	<0.0001	0.94	<0.0001	0.91	<0.0001	0.93	<0.0001
***HERC5***	0.81	<0.0001			0.91	<0.0001	0.91	<0.0001	0.95	<0.0001
***UBA1***	0.89	<0.0001	0.76	<0.0001			0.9	<0.0001	0.89	<0.0001
***UBC***	0.58	<0.0001	0.69	<0.0001	0.49	0.003			0.85	<0.0001
***USP18***	0.75	<0.0001	0.76	<0.0001	0.56	0.0005	0.54	0.0007		

Spearman's rho (r_s_) calculated by Spearman's rank correlation coefficient. r_s_ and *P* values in the lower-left area are the correlation of relative expression between genes in adjacent non-tumour tissues. r_s_ and *P* values in the upper-right area are the correlation of relative expression between genes in HCC tumour tissues.
